# Nonlinear age effects in tactile processing from early childhood to adulthood

**DOI:** 10.1002/brb3.2644

**Published:** 2022-06-08

**Authors:** Sakshi Kaur, Svenja Espenhahn, Tiffany Bell, Kate J. Godfrey, Chidera Nwaroh, Adrianna Giuffre, Lauran Cole, Winnica Beltrano, Tingting Yan, Mehak Stokoe, Logan Haynes, Tasha Yuntao Hou, Mark Tommerdahl, Signe Bray, Ashley D. Harris

**Affiliations:** ^1^ Department of Radiology Cumming School of Medicine University of Calgary Calgary Alberta Canada; ^2^ Alberta Children's Hospital Research Institute University of Calgary Calgary Alberta Canada; ^3^ Child and Adolescent Imaging Research (CAIR) Program University of Calgary Calgary Alberta Canada; ^4^ Hotchkiss Brain Institute University of Calgary Calgary Alberta Canada; ^5^ Department of Neuroscience University of Calgary Calgary Alberta Canada; ^6^ Department of Biomedical Engineering University of North Carolina at Chapel Hill Chapel Hill North Carolina USA

**Keywords:** development, discrimination thresholds, pediatric, somatosensory, tactile, vibrotactile

## Abstract

**Background:**

Tactile processing plays a pivotal role in the early stages of human development; however, little is known about tactile function in young children. An understanding of how tactile processing changes with age from early childhood to adulthood is fundamental in understanding altered tactile experiences in neurodevelopmental disorders, such as autism spectrum disorder.

**Methods:**

In this cross‐sectional study, 142 children and adults aged 3–23 years completed a vibrotactile testing battery consisting of 5 tasks, which rely on different cortical and cognitive mechanisms. The battery was designed to be suitable for testing in young children to investigate how tactile processing changes from early childhood to adulthood.

**Results:**

Our results suggest a pattern of rapid, age‐related changes in tactile processing toward lower discrimination thresholds (lower discrimination thresholds = greater sensitivity) across early childhood, though we acknowledge limitations with cross‐sectional data. Differences in the rate of change across tasks were observed, with tactile performance reaching adult‐like levels at a younger age on some tasks compared to others.

**Conclusions:**

While it is known that early childhood is a period of profound development including tactile processing, our data provides evidence for subtle differences in the developmental rate of the various underlying cortical, physical, and cognitive processes. Further, we are the first to show the feasibility of vibrotactile testing in early childhood (<6 years). The results of this work provide estimates of age‐related differences in performance, which could have important implications as a reference for investigating altered tactile processing in developmental disorders.

## Graphical Abstract

Tactile processing is essential in early child development and continues to develop throughout childhood and adolescence. Here, we examine the development of tactile performance across 5 tasks in 142 children and adults aged 3–23 years. All tasks show a pattern of rapid age‐related improvement that plateau by adulthood; however, there are pronounced differences in the rate of change. For example, we show a rapid decrease in reaction time (left) while simultaneous amplitude discrimination performance (right) develops more slowly.

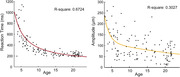

## 1 INTRODUCTION

Tactile perception plays a pivotal role in the early stages of human development. One of the primary means children use to explore and learn is through tactile experiences (Cascio, 2010; Narvaez et al., 2019). While understanding tactile processing is relevant to multiple clinical disorders, it is especially critical to investigate developmental disorders in which tactile abnormalities occur and can have substantial effects on everyday activities for children and adolescents, as seen in autism spectrum disorder, attention‐deficit hyperactivity disorder, and Tourette's syndrome (Little et al., 2018; Panagiotidi et al., 2018; Reynolds & Lane, 2009; Tomchek & Dunn, 2007). However, investigating tactile function in early childhood (< age 6 years) can be difficult.

Sensory processing in children has been investigated using various methods. Questionnaire‐based assessments—either self‐report or parent‐report—have been commonly used across a large age range to examine sensory function in healthy and clinical conditions (e.g., ASD, ADHD) (Jorquera‐Cabrera et al., 2017; Kamath et al., 2020; Kern et al., 2007; Leekam et al., 2007; Suzuki et al., 2019). However, questionnaires can only inform about aspects of sensory processing at the level of observable reactions. Among sensory assessments that measure the brain's functional response to sensory stimuli, electroencephalography (EEG), functional magnetic resonance imaging (fMRI), and magnetoencephalography (MEG) have been utilized to investigate the activity of various specific networks involved in sensory perception, as well as detect abnormalities in individuals with neurological disorders (Atagun et al., 2020; Bak et al., 2011; Demopoulos et al., 2017; Pierce et al., 2021; Schauder & Bennetto, 2016; Wang et al., 2014). In particular, discrimination tasks have been linked to GABAergic inhibition. In addition to its role in developmental plasticity, GABA (gamma‐aminobutyric acid) mediates lateral and feedforward inhibition (Ben‐Ari et al., 2012; Schmidt & Mirnics, 2015); higher GABA has been associated with lower tactile discrimination levels (i.e., greater sensitivity) (Puts et al., 2011; Puts, Wodka, et al., 2017; Tannan et al., 2008; Tommerdahl et al., 2010) due to better perceptual separation of stimuli (i.e., greater contrast) (Alloway & Burton, 1986; Dykes et al., 1984; Juliano et al., 1989). While neuroimaging studies provide valuable information about brain networks and neural responses to tactile stimuli, they do not characterize perceptual sensitivities. Furthermore, they are more reliable in older pediatric populations (i.e., age 6 years and above), as these methodologies may be difficult to perform in young children, and more so in children with behavioral and communication deficits (Larson & Taulu, 2017; O'Shaughnessy et al., 2008; Raschle et al., 2012).

Vibrotactile testing paradigms can quantitatively examine the function (or dysfunction) of somatosensory processing and can be designed to provide insight into multiple cortical functions (Mikkelsen et al., 2020; Nguyen et al., 2013; Puts et al., 2013, 2014; Tommerdahl et al., 2016). The different metrics have been shown to be sensitive to specific neurosensory mechanisms in humans, and parallels in said assessments have been observed in animal models (Tommerdahl et al., 2021). Furthermore, the relationship between some vibrotactile measures and their proposed targeted cortical mechanism have been supported through neuroimaging studies. For example, in adults, amplitude discrimination is informative regarding lateral inhibition between cortical regions and has been similarly demonstrated using fMRI (Maeda et al., 2013a, 2013b). Temporal order judgment has previously provided information on the functional connectivity of proximal cortical ensembles in the primary somatosensory cortex in adults (Tommerdahl et al., 2008). Duration discrimination (DD) has been shown to provide insight into the connection between timing perception and parietal‐cerebellar processing in adults (Bijsterbosch et al., 2011; Koch et al., 2007). Overall, vibrotactile testing is a convenient, noninvasive technique that can examine multiple specific mechanisms of sensory processing altogether, which we propose may be useful for studies in young children.

While previous tactile testing batteries have been developed for children, they have been validated only for children aged 8 years and older (Puts et al., 2013). Given that early childhood is a period of profound neurodevelopment (Dimond et al., 2020; Reynolds et al., 2019), it seems likely that the processing of tactile information changes across early childhood such that adult‐like task performance is seen by late childhood. Understanding how tactile processing changes with age across childhood and adolescence in typically developing children will establish a normative baseline for comparison in studies of neurodevelopmental conditions.

Here, we adapted and customized a vibrotactile battery (Puts et al., 2013) designed to examine multiple features of cortical processing and evaluated tactile performance cross‐sectionally across development in participants aged 3–23 years. We developed this customized vibrotactile testing battery to be appropriate for use in younger children aged 3–6 years. The large age range was selected to best examine tactile performance changes across development, from early childhood, across childhood and adolescence, to young adults. The lower age of 3 years was chosen as we expected this to be the youngest age in which we would be able to collect this data. As an upper age, we collected data into early adulthood (age 23 years), at which age we expect tactile development to have plateaued, while avoiding aging‐related changes or midlife clinical diagnoses that may influence tactile perception. Our battery included five tasks: Reaction time (RT), sequential and simultaneous amplitude discrimination (sqAD, smAD), temporal order judgment (TOJ), and duration discrimination (DD).

Using this newly developed tactile testing battery, in the current cross‐sectional study, we examine how thresholds for each of these tasks relate to age and demonstrate different nonlinear relationships between task performance and age. Thus, in addition to describing a vibrotactile testing battery that can be used in early childhood, this paper provides reference information regarding the age trajectories of these tasks, which may be used to inform future clinical research studies for new insight into understanding cortical function, how cortical functioning changes with development (and by extension, aging), and its alterations in clinical disorders.

## 2 MATERIALS AND METHODS

### 2.1 Participants

One hundred and forty‐two typically developing children and adults aged 3–23 years were recruited across four independent research studies at the University of Calgary. The four studies used the same vibrotactile testing approach but recruited different age ranges. The studies recruited: Early childhood (ages 3–6 years, *N* = 45 recruited), late childhood (ages 7–12 years, *N* = 34 recruited), adolescence (ages 13–17 years, *N* = 22 recruited), and adulthood (ages 18–23 years, *N* = 41 recruited) (Table 1). There were 7 pairs and 1 trio of siblings in the overall sample. All participants fulfilled the following inclusion criteria: no history of neurological, psychiatric, or neurodevelopmental disorders, no history of major head trauma, and no use of psychotropic medication. In accordance with the Declaration of Helsinki, prior written informed consent was obtained from either the participant or a parent of the participant (who themselves provided assent). Ethical approval for each study was obtained from the respective ethics board.

**TABLE 1:** Characteristics of study participants

	Early childhood	Late childhood	Adolescence	Adulthood	*p*‐value
N	45	34	22	41	
Age [years]	5.24 ± 1.16	10.10 ± 1.60	15.28 ± 1.28	20.88 ± 1.18	
Gender (M:F)	31:14	26:18	10:12	23:18	0.494
Handedness (R:L)	43:2	30:4	22:0	40:1	0.165

*Note*: Values given are means ± SD. Pearson Chi Square tests were used to test for group differences.

Abbreviations: M, male; F, female; R, right‐handed; L, left‐handed.

### 2.2 Experimental design

**FIGURE 1:**
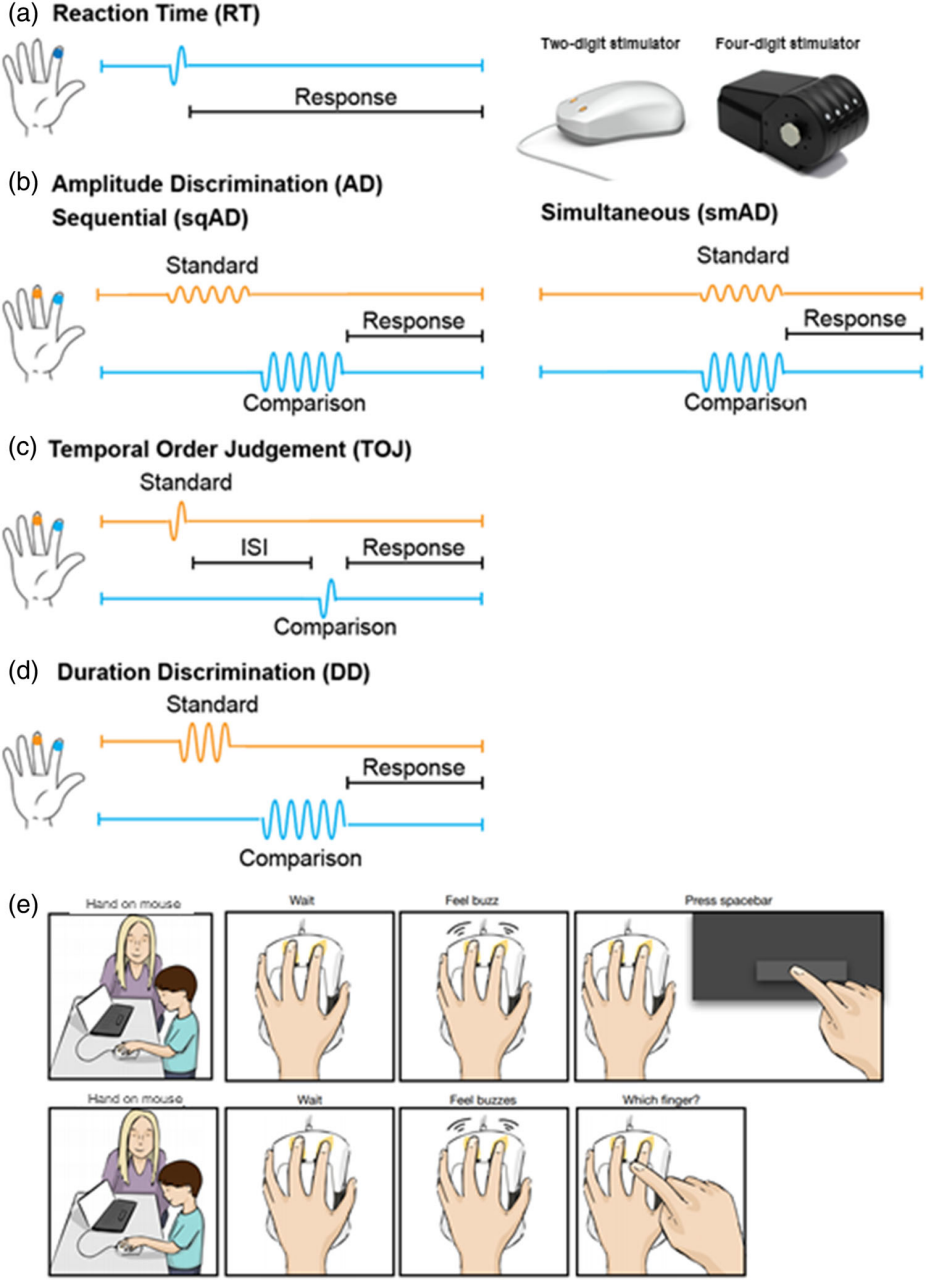
Schematic of vibrotactile testing battery. Participants completed a vibrotactile testing battery consisting of five different tasks using a Brain Gauge two‐digit or four‐digit stimulator (depicted top right). (a) reaction time (RT). (b) Sequential (sqAD) and simultaneous amplitude discrimination (smAD). (c) Temporal order judgment (TOJ). (d) Duration discrimination (DD). The standard stimulus is shown in orange and the comparison stimulus in blue for all tasks. (e) Visual aids were used to help young children understand task. The upper comic shows instructions for the RT task, while the lower comic shows instructions for the discrimination tasks.

All participants completed a visit to the Alberta Children's Hospital, which included vibrotactile psychophysical testing and other cognitive tasks related to the four independent studies. The vibrotactile testing battery consisted of five different tasks (Figure 1), with the order of the tasks being fixed, in line with previous studies (Puts et al., 2013; Tommerdahl et al., 2008). Vibrotactile stimuli were delivered to the participant's left index and middle finger using either a two‐digit or four‐digit tactile stimulator (Cortical Metrics, North Carolina, USA). All stimuli were in the flutter range (25–50 Hz), activating primary and secondary somatosensory cortex while avoiding high‐frequency vibrational frequency stimuli that are more related to secondary somatosensory cortex activation and shows transient cortical responses. Stimuli were delivered to the glabrous skin using cylindrical probes (5 mm diameter). In all tasks, stimulus delivery was pseudo‐randomized between the two fingers. A Google Chromebook or MacBook Pro running CM4 software (Holden et al., 2012) was used for data collection and to provide visual feedback.

Each task included at least three practice trials that required consecutive correct responses to proceed, confirming that participants understood the task and its goal. For the early childhood group, in addition to verbal instructions, visual aids were used (Figure 1e). For all participants, feedback was provided during practice trials but not during the task trials. Participants responded via a mouse‐click using their right hand. In the case of young children, for the reaction time task, they were asked to click the mouse when they felt the stimulus; however, for the other discrimination tasks, they pointed to the respective finger and the experimenter entered the response. As the response time was not relevant in the discrimination tasks, this was the best compromised to ensure data quality in this group. For all tasks, except reaction time, a staircase procedure was used to modulate the test parameter. Performance was assessed for each individual task; an individual task result was removed if it was three standard deviations away from the group mean. Additionally, task compliance was assessed for the four discrimination tasks; if a participant only selected one finger (did not switch at least 3 times across the 20 trials), it was interpreted that they did not understand the task and that task was excluded.

### 2.3 Vibrotactile tasks

#### 2.3.1 Reaction time (RT) and RT variability

Suprathreshold stimuli (frequency: 25 Hz; amplitude: 300 μm; duration: 40 ms) were delivered pseudo‐randomly to the left middle or index finger, and participants were asked to respond by either pressing the spacebar of the Chromebook or a button on a separate wired mouse with the right hand “as quickly as possible” upon feeling a stimulus (Figure 1a). For each participant, 10 trials with inter‐trial interval (ITI) of 4000–7000 ms were collected. A measure of reaction time was calculated by averaging over the median 6 trials (excluding the two fastest and slowest trials), which has been a standard reaction time calculation used in previous studies (Favorov et al., 2019; Nguyen et al., 2013; Puts et al., 2013, 2015, 2014; Zhang et al., 2011). Reaction time variability was also calculated as the standard deviation over these median six trials.

#### 2.3.2 Sequential and simultaneous amplitude discrimination (sqAD, smAD)

In the sequential amplitude discrimination (sqAD) task, two stimuli (both frequency: 25 Hz; duration: 500 ms) were delivered sequentially to the two fingers (interstimulus interval [ISI]: 500 ms), with one stimulus having a higher amplitude (Figure 1b). One finger received a standard stimulus (amplitude: 200 μm) and the other received a comparison stimulus with an initial stimulus amplitude of 400 μm. Participants were asked which finger received the higher amplitude stimulus. The comparison stimulus amplitude was decreased by 20 μm for each correct answer and increased by 20 μm for incorrect answers. In the smAD task, the procedure was identical, but the two stimuli were delivered simultaneously (20 trials total; ITI: 5000 ms). Amplitude discrimination thresholds for both tasks (sqAD and smAD) were calculated as the mean difference in amplitude between the standard and comparison stimulus of the final five trials. Participants with a smaller discrimination threshold on the amplitude discrimination tasks were able to successfully discriminate between stimuli which were closer in amplitude.

#### 2.3.3 Temporal order judgment (TOJ)

Two stimuli (both frequency: 25 Hz, amplitude: 300 μm, duration: 40 ms) were delivered to the left index and middle finger, separated temporally by a starting ISI of 150 ms (20 trials total; ITI: 5000 ms). The ISI was decreased by 15% for correct trials and increased by 15% for incorrect trials. Participants were asked to distinguish which finger received the first stimulus (Figure 1c). The temporal order judgment (TOJ) threshold was taken as the mean of the ISIs of the final five trials. Participants with a smaller discrimination threshold on the TOJ task were able to successfully discriminate between stimuli presented closer in time.

#### 2.3.4 Duration discrimination (DD)

In the duration discrimination (DD) task, two stimuli (both frequency: 40 Hz; amplitude: 300 μm) were delivered sequentially to the middle and index finger (ISI: 500 ms), with one finger receiving a longer duration stimulus (initial comparison stimulus duration: 750 ms; standard stimulus duration: 500 ms; 20 trials total; ITI: 5000 ms). The duration of the comparison stimulus was decreased by 25 ms for each correct answer and increased by 25 ms for each incorrect answer. Participants were asked which finger received the longer duration stimulus (Figure 1d). The DD threshold was obtained as the mean difference in duration of the standard and comparison stimulus for the final five trials. Participants with a smaller discrimination threshold on the DD task were able to successfully discriminate between stimuli which were more similar in duration.

### 2.4 Analysis

Data analysis was performed using custom‐written R (version 3.5.3; R Foundation for Statistical Computing, Vienna, Austria), MATLAB (version R2017b; The MathWorks, Inc., Natick, MA, USA), and SPSS (IBM SPSS Statistics for Windows, Version 25.0. Armonk, NY, USA) routines. Data for an individual task was excluded when it was reported by the experimenter that the task had not been executed properly (e.g., poor behavioral compliance) and/or visual inspection of the profile of the staircase showed large deviations from the expected profile (e.g., moving away from a threshold or only choosing one finger when responding), as may occur with fatigue or loss of focus.

### 2.5 Modeling polynomial age effects

Initially, to examine the relationship between age and task performance, linear models were developed and subsequently age^2^ and then age^3^ terms were added and evaluated using linear regression. The higher order terms were removed if they did not better explain the data. Results were considered significant if uncorrected *p*‐values were below 0.05. We subsequently modeled data with power function models that do not have any data distribution assumptions to better characterize nonlinear associations with age.

#### 2.5.1 Modeling other nonlinear age effects

For each task, individual vibrotactile measures were plotted as a function of age in years across groups. The polynomial models computed earlier were tested and compared with linear, exponential, and power models, where the model with the best fit is presented. The best fit for each individual task was determined by squared estimates of error (SSE), R‐square, and root mean square error (RMSE) values. The fitting approaches do not have any assumptions about the underlying data distribution. Final data models used were: exponential (*f*(*x*) = *a* × exp(*b* × *x*) + *c* × exp(*d* × *x*)) and power (*f*(*x*) = *a* × *x*^*b* + *c*) models.

#### 2.5.2 Task performance correlations

The relationships of performance across the different tasks may also provide insight into the development of tactile processing. In this exploratory analysis, task performance between all tasks was examined in a correlation matrix of the adult participants (age >18 years) under the assumption that the young adults in this study represent stabilized tactile performance development. A task performance correlation matrix was also formed for participants <18 years. To investigate the developmental trajectory of these task performance correlations, pairs of tasks were summarized as a ratio (e.g., RT/RTVar, sqAD/smAD, and TOJ/DD) and plotted against age. For the task pairs, RT was paired with all other tasks (RT/RTVar, RT/sqAD, RT/smAD, RT/TOJ, RT/DD) and then related tasks (amplitude discrimination tasks sqAD/smAD and timing‐related tasks TOJ/DD) were also visualized. These age relationships were assessed with linear regression.

## 3 RESULTS

### 3.1 Task completion

**FIGURE 2:**
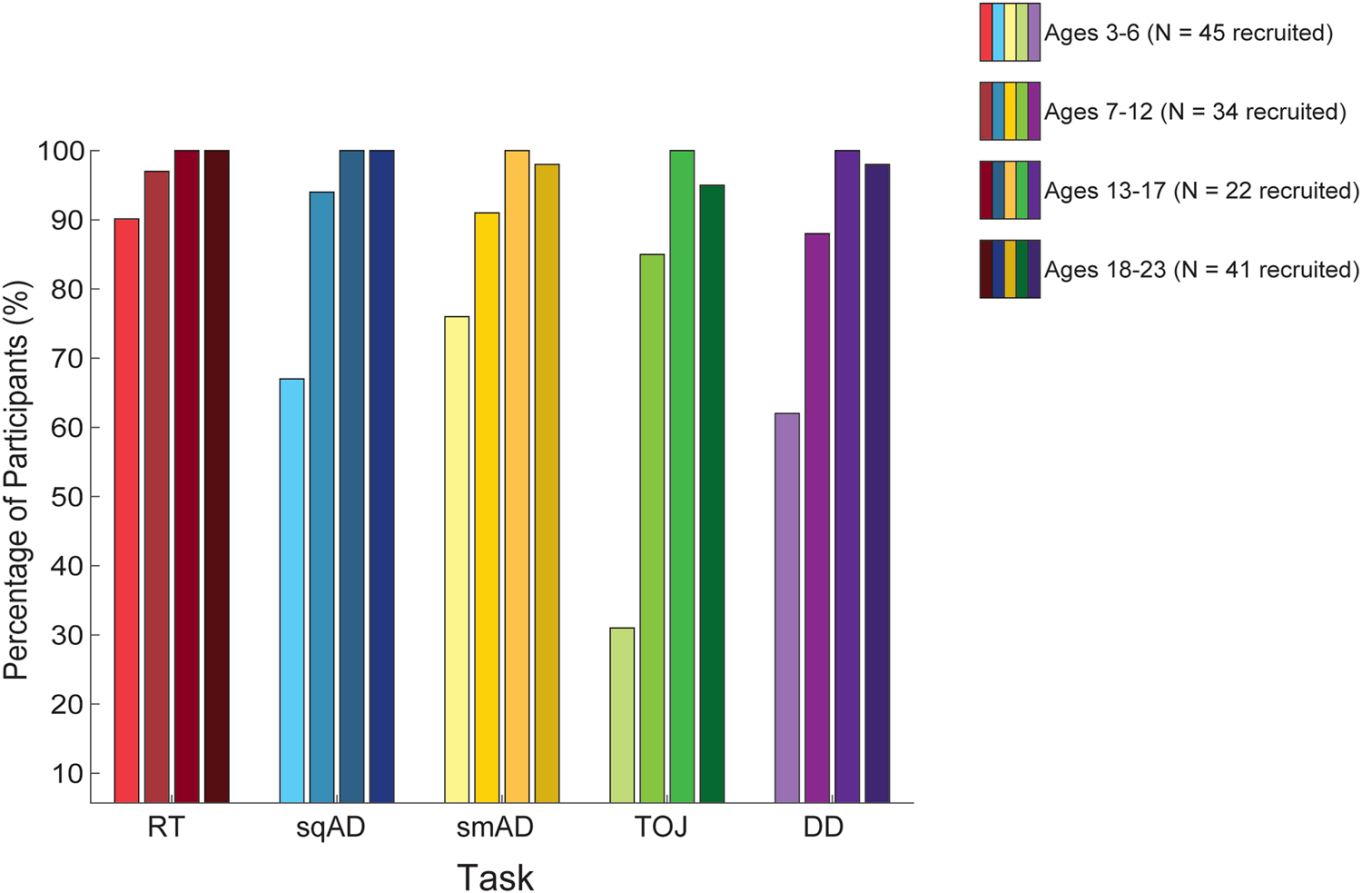
Task completion rate. Percentage of participants from each data collection group able to complete each vibrotactile task. RT: Reaction Time task (red); sqAD: sequential Amplitude Discrimination task (blue); smAD: simultaneous Amplitude Discrimination task (yellow); TOJ: temporal order judgment task (green); DD: duration discrimination task (purple). (Legend: Color shade indicates age group). See Table 2 for more information.

Compared to older participants, fewer participants in the early childhood data collection group were able to perform each task (Figure 2). The lower task completion rate included those who did not attempt a task, those who failed practice trials, and those who were excluded due to improper task completion. For example, some young children needed repeated prompting to press the spacebar upon stimulus delivery in the RT task, resulting in delayed responses. Therefore, RT data that were more than three standard deviations away from the group mean (for each age group) were excluded from this task and the reaction time variability calculations. To assess compliance for the amplitude discrimination (sqAD, smAD) and temporal discrimination (TOJ, DD) tasks, participants’ finger choices were assessed, alongside excluding mean thresholds that were three standard deviations from the group mean. As per the methods, participants who consistently answered with only one finger (and did not switch their response more than three times) were considered to have not understood the task or not complying and that task was excluded.

The data from three 3‐year‐old participants were completely excluded as they were judged by the experimenter to not have understood any of the vibrotactile tasks. Additional data exclusions by task are as follows: RT: *N* = 8 excluded (four participants aged 3 years, two participants aged 5 years, one participant aged 7 years, one participant aged 9); sqAD: *N* = 2 excluded (ages 3 and 5 years); TOJ: *N* = 4 excluded (three participants aged 5 years, one participant aged 6 years); DD: *N* = 5 excluded (two participants aged 4 years, two participants aged 5 years, one participant aged 6 years). Table 2 summarizes the initial number of participants recruited from each study and the final number of participants that were included in statistical analyses.

**TABLE 2:** Group sizes of recruited and included participants for each task

Age group		RT	sqAD	smAD	TOJ	DD
3–6	Recruited	45	45	45	45	45
	Included	33	30	34	14	28
7–12	Recruited	34	34	34	34	34
	Included	33	32	31	39	30
13–17	Recruited	22	22	22	22	22
	Included	22	22	22	22	22
18–23	Recruited	41	41	41	41	41
	Included	41	41	40	39	41

Exclusions = participants that did not complete the task + exclusions made by experimenter following data quality checking^*^

^*^RT exclusion: participant mean RT was more than 3 SD away from age group mean

^*^sqAD, smAD, DD, TOJ exclusions: participants that only answered with one finger (did not switch response).

As can be seen from Figure 2, among all vibrotactile tasks, the RT task (red) had the highest completion rate in young children, while the TOJ task (green) displayed the lowest completion rate (31%), suggesting that these were the easiest and most difficult tasks for younger children, respectively.

### 3.2 Task‐performance polynomial modeling with age factors

All tasks showed a significant relationship in performance with age. Most tasks were best explained with by models including age, age^2^, and age^3^ terms (RT, RTVar, sqAD, and DD), while smAD was best modeled with only a linear age term and TOJ was best modeled with age and age^2^ terms. The final linear model and goodness of fit measures are summarized in Table 3. The individual model parameters and the plots of best model are included in the Appendix Table 1 and Appendix Figure 1.

**TABLE 3:** Polynomial model equations and goodness of fit measures

Measure	Model equation	*R*	*R* ^2^
RT	*y* = 1607.534+(–262.420)*x+16.416*x ^2^+(–0.335)*x ^3^	0.867	0.749
RTVar	*y* = 356.093+(–70.032)*x+4.713*x ^2^+(–0.102)*x ^3^	0.735	0.540
sqAD	*y* = 335.310+(–56.651)*x+3.680*x ^2^+(–0.077)*x ^3^	0.555	0.309
smAD	*y* = 170.984+(–5.073)*x	0.477	0.227
TOJ	*y* = 176.406+(–13.164)*x+0.311*x ^2^	0.565	0.319
DD	*y* = 508.145+(–77.298)*x+4.728*x ^2^+(–0.096)*x ^3^	0.691	0.478

### 3.3 Exponential and power age effects on vibrotactile measures

**FIGURE 3:**
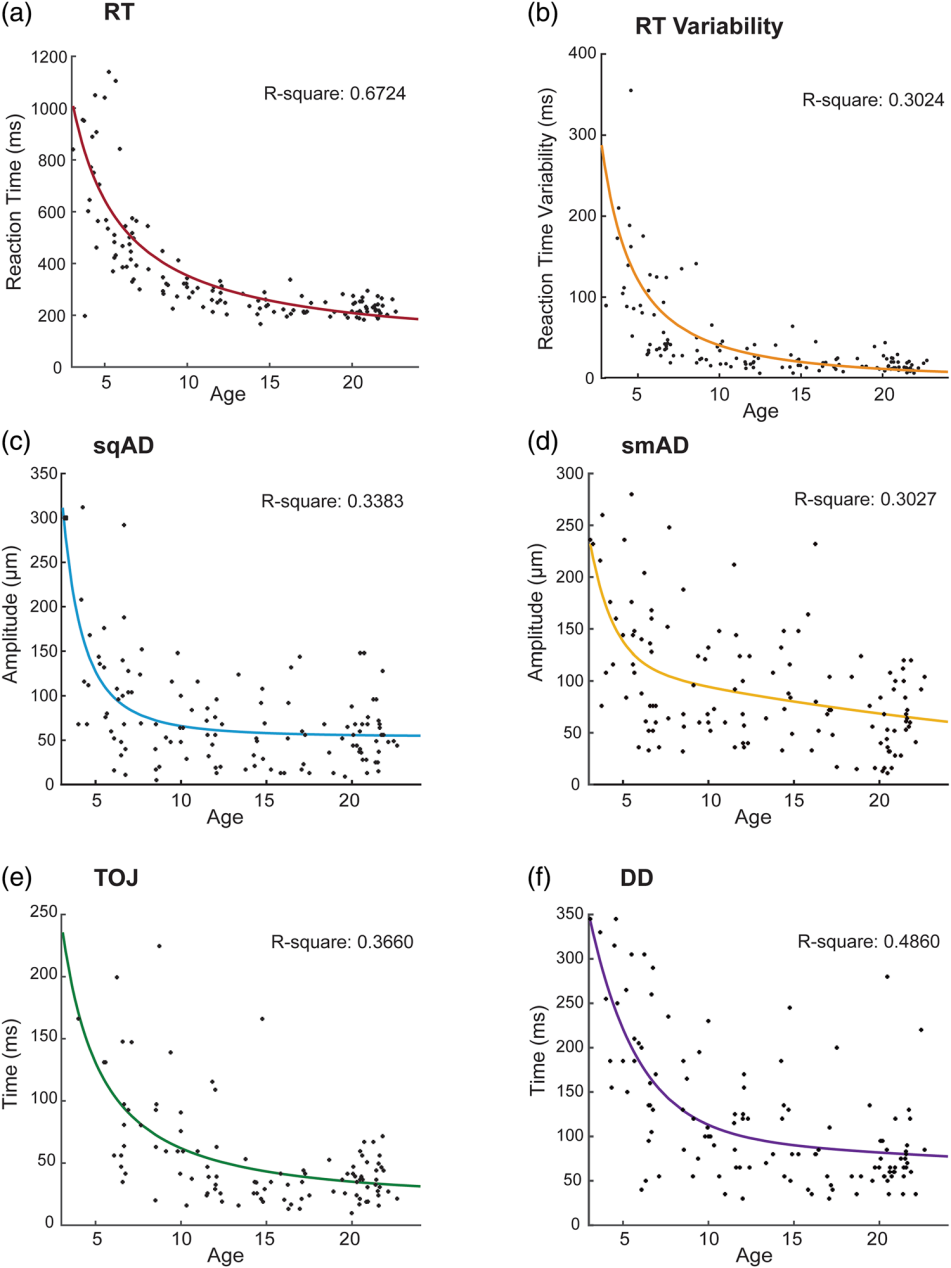
Exponential and power age effects in tactile processing from early childhood to adulthood. Nonlinear models were fit for each task as a function of age. Data points represent individual participants and curves represent best model fits. (a) Reaction time (ms) using a power fit. (b) Reaction time variability (ms) using a power fit. (c) Sequential amplitude discrimination (μm) using a power fit. (d) Simultaneous amplitude discrimination (μm) using an exponential fit. (e) Temporal order judgment (ms) using a power fit. (f) Duration discrimination (ms) using an exponential fit.

Power and exponential models showed the best fits of the age versus performance for each task (Figure 3). A power model best represented the age‐related changes in RT, RT variability, sqAD, and TOJ thresholds, and an exponential model best fitted the observed improvement in smAD and DD thresholds (Table 4). A rapid initial improvement in performance (i.e., decrease in threshold) was observed for each task, which then slows to a plateau. The sqAD, smAD, and DD thresholds improved rapidly during early childhood and plateaued around age 8–10 years. RT and TOJ thresholds, however, kept improving across late childhood and plateaued around age 15–17 years. While RT and TOJ share very similar curves in terms of rate of development, it is relevant to consider the task completion rate for RT was much higher for young children (96%) compared to TOJ (31%).

**TABLE 4:** Exponential and power models and goodness of fit measures

Task	Model equation	SSE	RMSE	R^2^
RT	f(x) = 3158(x)^–1.137^+108.2	2.034e + 06	124.6	0.674
RT variability	f(x) = 1884(x)^–1.573^ ‐ 7.658	1.125e + 06	93.02	0.302
sqAD	f(x) = 4767(x)^–2.591^+53.76	2.582e + 05	46.19	0.338
smAD	f(x) = 127.9e^–0.031*x^+1106e^–0.7311*x^	3.021e + 05	49.76	0.303
TOJ	f(x) = 1004(x)^–1.357^+17.8	1.098e + 05	33.14	0.366
DD	f(x) = 726.3e^–0.355*x^ + 104.6e^–0.013*x^	3.746e + 05	57.07	0.486

Abbreviations: SSE, squared estimates of error; RMSE, root mean square error.

#### 3.3.1 Task performance correlations

**FIGURE 4:**
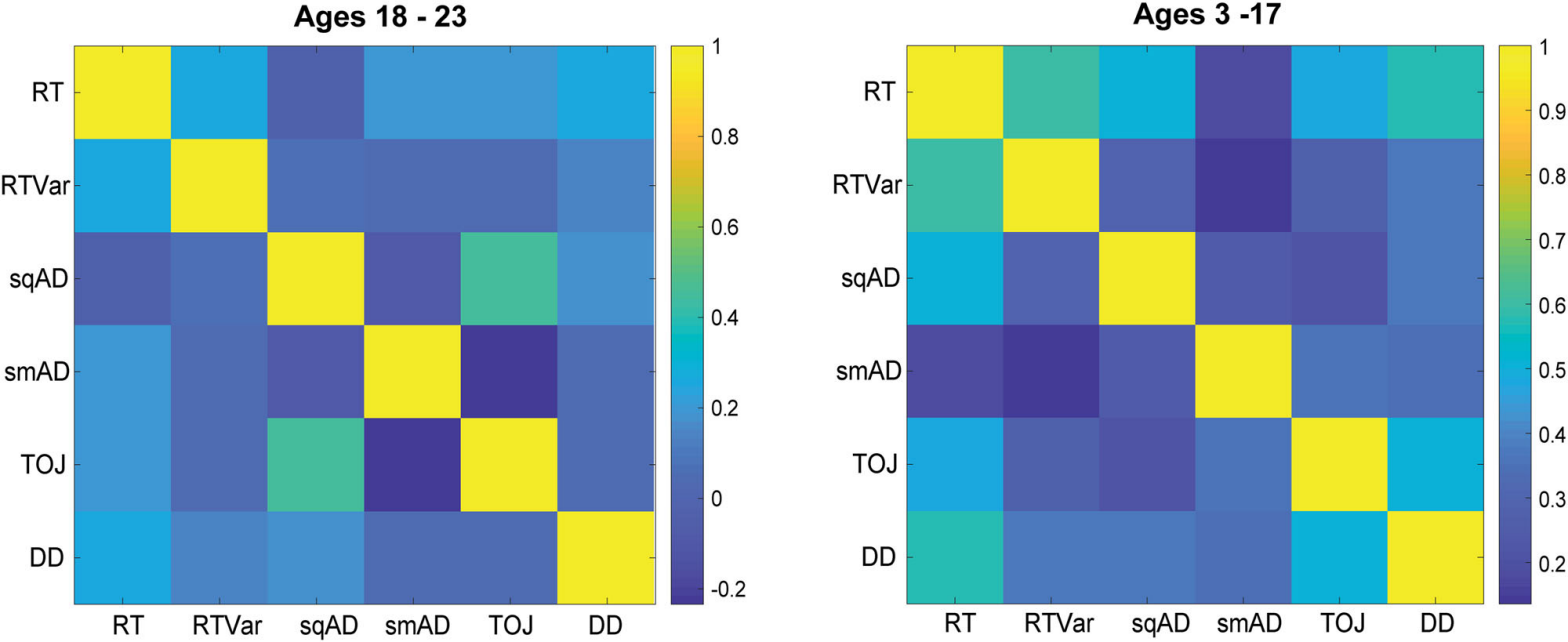
Correlation heatmap depicting relationship between each vibrotactile task compared to every other vibrotactile task. Dark blue indicates a weak correlation while yellow indicates a strong correlation. Heatmap on left displays task performance correlations in ages 18–23 years, while heatmap on right displays task performance correlations in ages 3–17 years.

**FIGURE 5:**
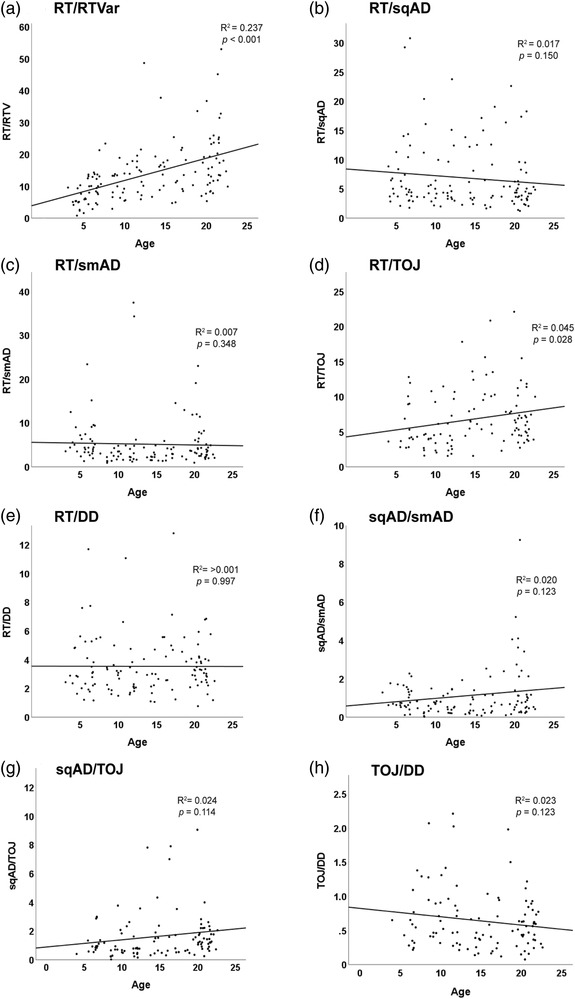
Age‐related changes in the performance of pairs of tasks. Linear models were fitted for each task pair as a function of age. Data points represent individual participants. (a) Reaction time/Reaction time variability. (b) Reaction time/Sequential amplitude discrimination. (c) Reaction time/Simultaneous amplitude discrimination. (d) Reaction time/Temporal order judgment. (e) Reaction time/Duration discrimination. (f) Sequential amplitude discrimination/Simultaneous amplitude discrimination. (g) Sequential amplitude discrimination/Temporal order judgment. (h) Temporal order judgment/Duration discrimination

The correlation matrix of task performance in the adult subgroup showed no tasks to be correlated with the exception of sqAD and TOJ, *r* = 0.45, *p* = 0.004, see Figure 4. By contrast, almost all tasks were correlated in the rest of the cohort, (i.e., participants aged 3–17 years). Figure 5 shows the relationship of the performance ratio in pairs of tasks with age. Only RT/RTVar and RT/TOJ showed significant age relationships, although RT/TOJ would not survive correction for multiple comparisons.

## 4 DISCUSSION

In the present study, we explored age‐related changes in various measures of tactile processing from early childhood to adulthood using a vibrotactile testing battery that includes five different tasks. Although vibrotactile testing has been extensively used in older children and adults (Hanley et al., 2019; Puts et al., 2013; Puts, Wodka et al., 2017; Zhang et al., 2011), we are the first to quantitatively assess vibrotactile discrimination thresholds in young children under 6 years of age while expanding on existing findings about tactile processing in adolescence and adulthood (Bleyenheuft et al., 2006; Puts et al., 2013). This initial work not only demonstrates the feasibility of performing objective tactile testing in young children, but it also provides new understanding of tactile perception and its development. It lays the groundwork for future longitudinal studies to more thoroughly investigate developmental trajectories as well as developmental disorders, particularly those known to affect tactile processing and/or behavior.

Briefly, we examined the relationships between age and task performance using two approaches: First a more constrained model approach with age, age^2^, and age^3^ terms and secondly a more flexible approach including linear, power, and exponential models. Given the flexibility of the second approach, it is perhaps unsurprising this approach resulted in the best age‐related data fitting. Overall, our assessments show age‐related decline in vibrotactile thresholds (e.g., improvements in tactile sensitivity), with rapid changes occurring across early childhood, suggesting that this is an important period for tactile processing development. For some tasks, such as sqAD and smAD as well as DD, tactile performance reaches adult‐like levels in late childhood (ages 8–10 years). For other tasks, such as reaction time (RT) and TOJ, performance continues to improve until later in adolescence (ages 15–17 years). The pattern of rapid changes in vibrotactile thresholds during early childhood is likely a reflection of the profound brain development that occurs during this period (Gilmore et al., 2018; Lebel & Deoni, 2018), but the differences across tasks in terms of when adult‐like performance is reached suggests that the underlying cortical, physical, and cognitive processes develop at different rates. Indeed, each task reflects different aspects of cortical function and/or the integration of multiple functions. For example, a temporal order judgment tactile task not only relies on the perception and discrimination of two stimuli, but it also requires higher‐order processing and memory to determine which came first and last and elicit a response (M. Tommerdahl et al., 2019).

Unsurprisingly, young children had the slowest reaction times, which improved (i.e., decreased) with age to reach adult‐like levels during late childhood. The same pattern was observed in reaction time variability. This pattern is consistent with the proposed U‐shaped relationship between age and reaction time across the lifespan; increases in age throughout childhood are associated with decreases in reaction time and its variability, while increases in age throughout adulthood are associated with greater reaction time and its variability (Williams et al., 2005). The rapid improvement in reaction time seen during early childhood may be associated with the rapid white matter development, such as myelination and/or axonal growth that occurs in early childhood (Dimond et al., 2020; Mabbott et al., 2006; Reynolds et al., 2019; Scantlebury et al., 2014). Increased myelination will result in more efficient signal transduction, allowing for a faster reaction time (Chevalier et al., 2015).

For both simultaneous and sequential amplitude discrimination tasks (smAD, sqAD), rapid improvement (decrease) in discrimination thresholds was observed, with adult‐like levels reached in late childhood. On average, smAD thresholds were higher than sqAD thresholds, which can be explained by the greater difficulty of the former task. GABAergic lateral inhibition has been shown to play a pivotal role in separating tactile stimuli (Alloway & Burton, 1986; Dykes et al., 1984; Juliano et al., 1989), and higher GABA levels have been related to lower tactile discrimination thresholds (higher sensitivity) (Puts et al., 2011; Puts, Wodka et al., 2017; Tannan et al., 2008; Tommerdahl et al., 2010). As many developmental disorders have been associated with altered GABAergic inhibition, it is perhaps not surprising that links between tactile behaviors and altered GABA have been demonstrated (Puts et al., 2011, 2015; Puts, Harris, et al., 2017; Puts, Wodka et al., 2017; Tavassoli et al., 2016). Based on previous in vivo findings of age‐related GABA changes in childhood (Porges et al., 2021; Saleh et al., 2020), we speculate that our behavioral findings may, by extension, reflect age‐related changes in inhibitory function with development.

Similar to amplitude discrimination thresholds, DD thresholds appear to be adult‐like by late childhood. We again speculate that increases in GABAergic inhibition during childhood improves the contrast of neuronal responses to tactile stimuli, which results in increased timing sensitivity in both temporal discrimination tasks (DD and TOJ). GABA mediates lateral and feed‐forward inhibition (Ben‐Ari et al., 2012; Schmidt & Mirnics, 2015), with higher GABAergic inhibition being associated with lower tactile discrimination thresholds (i.e., greater sensitivity) (Puts et al., 2011; Puts, Wodka et al., 2017; Tannan et al., 2008; Tommerdahl et al., 2010) due to better perceptual separation of stimuli (i.e., greater contrast) (Alloway & Burton, 1986; Dykes et al., 1984; Juliano et al., 1989). Additionally, cortical grey matter development progresses in a parietal‐to‐frontal (posterior‐to‐anterior) pattern (Gogtay et al., 2004; Teffer & Semendeferi, 2012). As the parietal cortex, with engagement from the cerebellum (Dormal et al., 2016; A. P. Tommerdahl et al., 2019), has been suggested to be involved in processing of duration information, its early maturation relative to prefrontal regions is in accordance with earlier maturation of DD relative to TOJ thresholds.

TOJ thresholds reached adult‐like levels later than other discrimination thresholds. Growing evidence suggests the prefrontal cortex plays an integral role in temporal ordering (Lee et al., 2013; Pastor et al., 2004; Takahashi et al., 2013), and lesions in the frontal lobe have been associated with impaired temporal discrimination (Koch et al., 2009; Lacruz et al., 1991). The later maturation of the prefrontal cortex (Gogtay et al., 2004; Luna et al., 2015; Teffer & Semendeferi, 2012) may explain the late plateau in TOJ thresholds. Interestingly, few of the 3‐ and 4‐year‐olds were able to successfully complete the TOJ task. Previous studies have shown that 3‐year‐old children have more difficulties understanding and using temporal terms compared to 5‐year‐old children (Busby Grant & Suddendorf, 2011), and that temporal memory is linked to the understanding of temporal terms (McColgan & McCormack, 2008). Hence, it may be that young children in our study had difficulties with the language used to explain the task goal (i.e., understanding the terms “first” and “second”). Notably, a larger proportion of the young children were able to complete the DD task compared to the (TOJ) task and we therefore suggest that fatigue alone due to the length of the testing does not explain the apparent great difficulty with the temporal order judgment task. Temporal order judgment being the most difficult task for children is not completely unexpected, as temporal ordering has been previously observed to be the most difficult task for adults as well (Love et al., 2013).

The task performance correlation matrices suggest that in adults, among the tasks performed in this battery, the only correlated task performance is between sqAD and TOJ. The common element of these tasks is presentation of similar stimuli at different times (sequentially). As such there is a common working memory task in these two perceptual tasks. Interestingly, the task correlation matrix including the participants under 18 showed multiple correlated measures. When examining how age affects the relationships between paired tasks, a couple age‐related associations of paired task performance were found. RT/RTVar showed a strong age‐relationship; however, these two measures are from the same task and thus are interrelated. RT/TOJ also showed age‐related changes, but this was a weak effect, and the significance would not survive correction for multiple comparisons. We therefore suggest that task performance correlations that are seen when pooling across age actually reflect the rapid task performance improvements across childhood and not that relationships in task performance deteriorates with age.

Multiple factors that may affect performance across discrimination tasks and particularly relevant in this developmental study are working memory, attention, and the development of related brain networks. For example, the frontoparietal and salience networks are engaged by demanding tasks and develop profoundly throughout childhood and adolescence. As such, working memory also matures across this age range (Klingberg et al., 2002). In all discrimination tasks except smAD, participants had to maintain the first stimulus in their memory to compare it to the second stimulus. Considering children have lower working memory capacities than adults (Pelegrina et al., 2015; Tamnes et al., 2013), and existing evidence has shown that working memory improves linearly with age from early childhood to late adolescence (Gathercole et al., 2004; Pelegrina et al., 2015; Tamnes et al., 2013), we suggest the working memory requirements for these tasks may be met by late childhood, allowing successful task performance. Assessing the effects of working memory on task performance may be examined in a future study by altering the interstimulus interval of the stimuli presented during discrimination tasks. Young children are also more distractible and have difficulties orienting and executing attention toward a task for extended periods of time (Lewis et al., 2018; Yan et al., 2018). Decreased attentiveness in children may have also contributed to the age‐related differences in tactile performance and explains the greater variability.

Our study has several strengths, including the introduction of a battery useful for vibrotactile testing in young children. The high completion rates, even among young children, supports the utility of our design. However, a few limitations are worth discussing in more detail. The sample size is small and thus this is a preliminary study of this tactile battery in young children. Additional validation and reliability studies in young children are needed as it is unknown whether the reliability as established in older groups (Mikkelsen et al., 2020) extends to childhood. A common limitation in studying pediatric cohorts is their compliance with task requirements. Our battery was relatively short (∼20 min) and psychophysical assessments are typically lengthier with more trials. Hence, we cannot rule out that our shorter battery did not affect measurement accuracy. However, increasing the number of trials, and thus overall testing time, could also adversely affect compliance and fatigue, and ultimately adversely affect measurement robustness. Additionally, our findings are based on cross‐sectional data, which limits our ability to draw developmental inferences. Other studies examining the effects of age have shown over‐estimation of effects in cross‐sectional data compared to longitudinal data, indicating the desire to perform follow‐up longitudinal studies. The data that was acquired across multiple studies also resulted in different response modalities being compiled together. As such, there is a chance of differential executive loading in the different age groups, which may influence developmental differences in vibrotactile performance. Furthermore, because data was acquired across multiple studies, we do not have socioeconomic data in all participants. It is well established that socioeconomic factors impact child development, but we are unable to assess or report those impacts in the current study. In the future, a longitudinal study including neuroimaging may describe developmental perceptual changes and their underlying cortical mechanisms.

In conclusion, our study is the first to show that different measures of tactile processing can be measured quantitatively in children under 6 years of age, and that performance of these tactile tasks improves exponentially with age from early childhood into adulthood. Each task in this vibrotactile battery provides complementary information that is weighted toward different aspects of processing tactile information—together, the tasks can provide a comprehensive overview on tactile function. Moving forward, these results provide a basis to investigate interindividual developmental trajectories to better understand typical heterogeneity and the context of other ongoing development, for example, the co‐occurring development of cognitive functions. Alternatively, these findings could be used as a reference to understand aberrant developmental trajectories seen in developmental disorders and in particular, this testing battery may be used to investigate early changes in tactile processing (i.e., early childhood). This may assist to define therapeutic strategies that are targeted by developmental stage. Future investigations of tactile processing in early childhood with neuroimaging and longitudinal assessments could also provide new insight into the neural basis of these age‐related effects and alterations in clinical disorders.

## CONFLICT OF INTEREST

Mark Tommerdahl is co‐founder of Cortical Metrics, LLC. Cortical Metrics is licensed by the University of North Carolina to distribute the tactile stimulator used in this study.

### PEER REVIEW

The peer review history for this article is available at https://publons.com/publon/10.1002/brb3.2644

## Supporting information

Supporting information

## Data Availability

The data supporting the findings in this study are available from Svenja Espenhahn upon reasonable request.
