# Supplementary material for: Nonlinear age effects in tactile processing from early childhood to adulthood
Source: Brain Behav. 2022 Jun 8;12(7):e2644. doi: 10.1002/brb3.2644 (PMC9304836; doi:10.1002/brb3.2644)
Supplement: Supplementary file 1 — Supporting information [file BRB3-12-e2644-s001.pdf]

## Supplementary Material

**Appendix Table 1.** Polynomial age model coefficients and significance measures.

| ***Task*** | ***Model*** | ***β*** | ***Std. Error*** | ***t*** | ***p-value*** |
|---|---|---|---|---|---|
| *RT* | Constant | 1607.534 | 117.249 | 13.710 | >0.001 |
| | Age | -262.420 | 34.381 | -7.633 | >0.001 |
| | $Age^2$ | 16.416 | 2.932 | 5.599 | >0.001 |
| | $Age^3$ | -0.335 | 0.075 | -4.465 | >0.001 |
| *RTVar* | Constant | 821.030 | 114.044 | 7.199 | >0.001 |
| | Age | -217.991 | 33.441 | -6.519 | >0.001 |
| | $Age^2$ | 19.117 | 2.852 | 6.703 | >0.001 |
| | $Age^3$ | -0.501 | 0.073 | -6.856 | >0.001 |
| *sqAD* | Constant | 335.310 | 55.731 | 6.017 | >0.001 |
| | Age | -56.651 | 15.842 | -3.576 | >0.001 |
| | $Age^2$ | 3.680 | 1.328 | 2.772 | 0.006 |
| | $Age^3$ | -0.077 | 0.034 | -2.281 | 0.024 |
| *smAD* | Constant | 170.984 | 12.136 | 14.089 | >0.001 |
| | Age | -5.073 | 0.833 | -6.088 | >0.001 |
| *TOJ* | Constant | 176.406 | 28.529 | 6.184 | >0.001 |
| | Age | -13.164 | 4.438 | -2.966 | 0.004 |
| | $Age^2$ | 0.311 | 0.154 | 2.024 | 0.046 |
| *DD* | Constant | 508.145 | 69.673 | 7.293 | >0.001 |
| | Age | -77.298 | 19.987 | -3.867 | >0.001 |
| | $Age^2$ | 4.728 | 1.684 | 2.807 | 0.006 |
| | $Age^3$ | -0.096 | 0.043 | -2.241 | 0.027 |

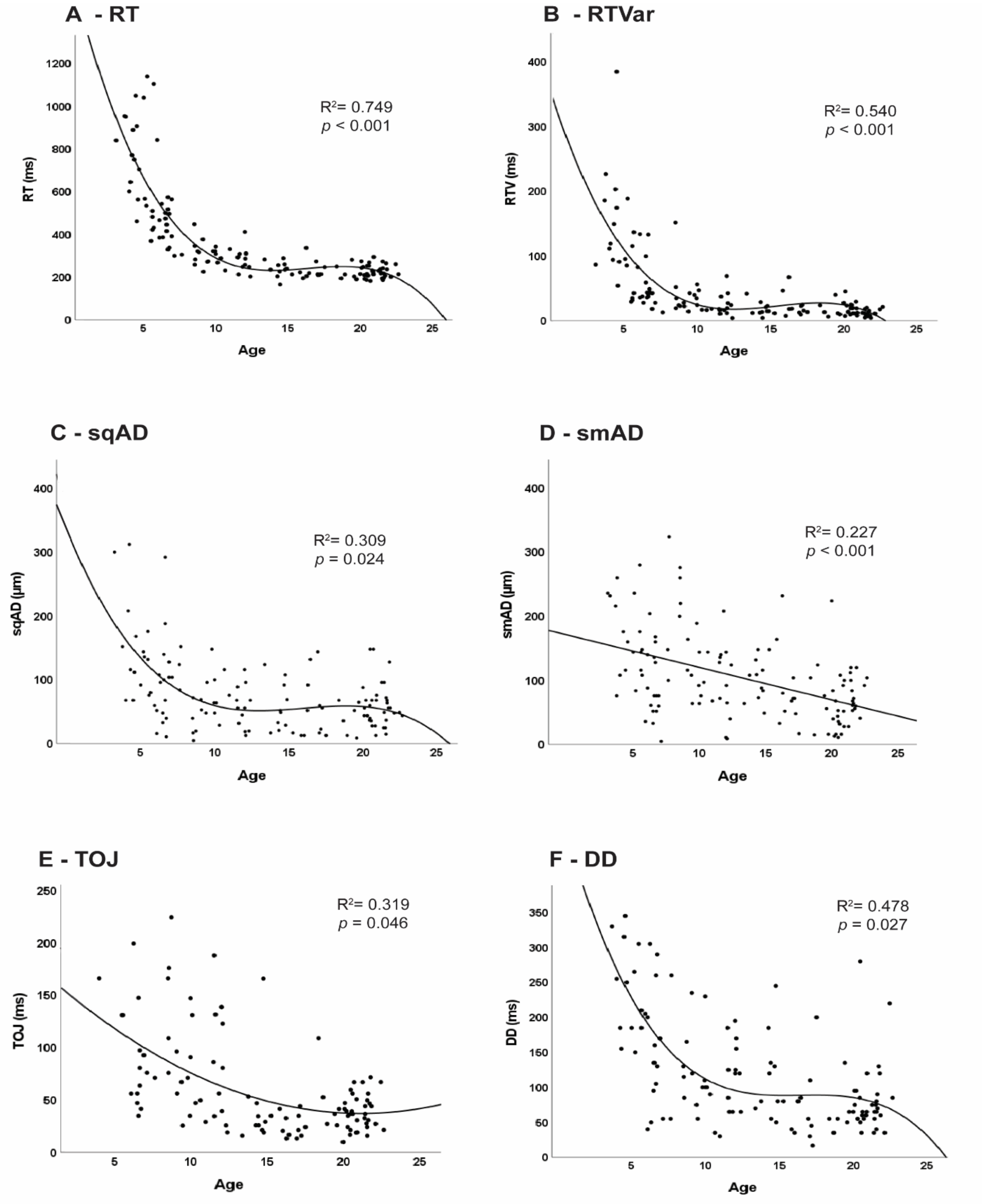


**Appendix Figure 1. Polynomial age effects in tactile processing from early childhood to adulthood.** Polynomial models were fitted for each task as a function of age. Data points represent individual participants and curves represent best fitted models. **A**, Reaction time (ms) **B**, Reaction time variability (ms) **C**, Sequential amplitude discrimination (µm) **D**, Simultaneous amplitude discrimination (µm) **E**, Temporal order judgement (ms) **F**, Duration discrimination (ms).

**Appendix Table 2**. Task pair ratios and age models coefficients and significance measures.

| ***Task Ratio*** | ***Model*** | ***β*** | ***Std. Error*** | ***t*** | ***p-value*** |
|---|---|---|---|---|---|
| *RT/RTVar* | Constant | 4.852 | 1.567 | 3.097 | 0.002 |
| | Age | 0.696 | 0.109 | 6.370 | >0.001 |
| *RT/sqAD* | Constant | 9.436 | 1.610 | 5.860 | >0.001 |
| | Age | -0.157 | 0.108 | -1.448 | 0.150 |
| *RT/smAD* | Constant | 7.032 | 1.636 | 4.299 | >0.001 |
| | Age | -0.104 | 0.110 | -0.942 | 0.348 |
| *RT/TOJ* | Constant | 4.492 | 1.107 | 4.058 | >0.001 |
| | Age | 0.157 | 0.070 | 2.235 | 0.028 |
| *RT/DD* | Constant | 3.551 | 0.454 | 7.821 | >0.001 |
| | Age | -0.001 | 0.030 | -0.029 | 0.977 |
| *sqAD/smAD* | Constant | 0.635 | 0.338 | 1.876 | 0.063 |
| | Age | 0.035 | 0.022 | 1.552 | 0.123 |
| *sqAD/TOJ* | Constant | 0.893 | 0.496 | 1.799 | 0.075 |
| | Age | 0.050 | 0.031 | 1.594 | 0.114 |
| *TOJ/DD* | Constant | 16.168 | 0.951 | 17.007 | >0.001 |
| | Age | -1.911 | 1.228 | -1.556 | 0.123 |
